# Multi-scale processes of beech wood disintegration and pretreatment with 1-ethyl-3-methylimidazolium acetate/water mixtures

**DOI:** 10.1186/s13068-015-0422-9

**Published:** 2016-01-08

**Authors:** Jörn Viell, Hideyo Inouye, Noemi K. Szekely, Henrich Frielinghaus, Caroline Marks, Yumei Wang, Nico Anders, Antje C. Spiess, Lee Makowski

**Affiliations:** Aachener Verfahrenstechnik-Process Systems Engineering, RWTH Aachen University, Turmstr. 46, 52064 Aachen, Germany; JARA-ENERGY, Jülich, Germany; Department of Electrical and Computer Engineering, College of Engineering, Northeastern University, 360 Huntington Avenue, Boston, MA 02115 USA; Jülich Centre for Neutron Science, Forschungszentrum Jülich GmbH, Outstation at MLZ, Lichtenbergstraße 1, 85747 Garching, Germany; Aachener Verfahrenstechnik-Enzyme Process Technology, RWTH Aachen University, Worringer Weg 1, 52074 Aachen, Germany; DWI-Leibniz Institute für Interactive Materials, Forckenbeckstr. 40, 52072 Aachen, Germany; Bioengineering Department and Chemistry and Chemical Biology Department, Northeastern University, 360 Huntington Ave., Boston, MA 02115 USA; Institute of Biochemical Engineering, Technische Universität Braunschweig, Gaußstr. 17, 38102 Braunschweig, Germany

**Keywords:** Ionic liquid, Lignocellulose, EMIMAc, XRD, SANS, Pretreatment, Crystallinity, Disintegration, Microfibrils

## Abstract

**Background:**

The valorization of biomass for chemicals and fuels requires efficient pretreatment. One effective strategy involves the pretreatment with ionic liquids which enables enzymatic saccharification of wood within a few hours under mild conditions. This pretreatment strategy is, however, limited by water and the ionic liquids are rather expensive. The scarce understanding of the involved effects, however, challenges the design of alternative pretreatment concepts. This work investigates the multi length-scale effects of pretreatment of wood in 1-ethyl-3-methylimidazolium acetate (EMIMAc) in mixtures with water using spectroscopy, X-ray and neutron scattering.

**Results:**

The structure of beech wood is disintegrated in EMIMAc/water mixtures with a water content up to 8.6 wt%. Above 10.7 wt%, the pretreated wood is not disintegrated, but still much better digested enzymatically compared to native wood. In both regimes, component analysis of the solid after pretreatment shows an extraction of few percent of lignin and hemicellulose. In concentrated EMIMAc, xylan is extracted more efficiently and lignin is defunctionalized. Corresponding to the disintegration at macroscopic scale, SANS and XRD show isotropy and a loss of crystallinity in the pretreated wood, but without distinct reflections of type II cellulose. Hence, the microfibril assembly is decrystallized into rather amorphous cellulose within the cell wall.

**Conclusions:**

The molecular and structural changes elucidate the processes of wood pretreatment in EMIMAc/water mixtures. In the aqueous regime with >10.7 wt% water in EMIMAc, xyloglucan and lignin moieties are extracted, which leads to coalescence of fibrillary cellulose structures. Dilute EMIMAc/water mixtures thus resemble established aqueous pretreatment concepts. In concentrated EMIMAc, the swelling due to decrystallinization of cellulose, dissolution of cross-linking xylan, and defunctionalization of lignin releases the mechanical stress to result in macroscopic disintegration of cells. The remaining cell wall constituents of lignin and hemicellulose, however, limit a recrystallization of the solvated cellulose. These pretreatment mechanisms are beyond common pretreatment concepts and pave the way for a formulation of mechanistic requirements of pretreatment with simpler pretreatment liquors.

**Electronic supplementary material:**

The online version of this article (doi:10.1186/s13068-015-0422-9) contains supplementary material, which is available to authorized users.

## Background

Cellulose makes up much of the material in lignocellulosic cell walls and has the potential to provide source for fuels, materials and chemicals. Mild and selective conversion is, however, difficult due to the composition and structure of cell walls, in which cellulose is packed in dense, fibrillar structures. The fibrils exhibit crystalline-like order that is maintained by intermolecular hydrogen bonds and they are cross-linked and embedded in a matrix of lignin and hemicellulose. Since this arrangement creates a major obstacle to enzymatic hydrolysis and efficient conversion, numerous pretreatment strategies for breakdown of the composite material have been developed to deconstruct the composite material [1, 2]. Nevertheless, these concepts have only been applied successfully to a limited range of biomass species and less so to the highly lignified wood. It demonstrates that the underlying mechanisms of pretreatment have not yet been fully understood.

The molecular and structural analysis by Langan et al. [3] revealed two common fundamental processes, that is, dehydration of cellulose fibrils and then phase separation of cleaved lignin-hemicellulose. The latter process dissolves lignin and hemicellulose from the cellulose matrix and a change of the solvent state possibly leads to subsequent precipitation of lignin globules on the fiber surface (as observed in [4]). In fact, a strong correlation is suggested between enzymatic digestibility and the lignin content in the cell wall [5] but other studies also mention the crystallinity of the cellulose to be limiting [2]. While lignin was regarded as an indirect barrier covering xylan that itself restricts the access to cellulose, porosity—in particular on the nm-scale—was also found to be highly involved [6].

Consequently, enzymatic digestibility is assigned to several parameters which depend on composition, but a lot more on ultrastructure [7]. The mere removal of matrix polymers cannot be advantageous in terms of access because the created voids seem too small for enzymes [8], which draws the attention to the remaining fibrillar network of cellulose with its crystalline structure and the macrofibrils at the nm-scale. The microfibrils of approximately 0.4 nm diameter contain the cellulose in the crystalline lattice called Iβ. These fibrils are organized in macrofibrils of 14–23 nm diameter [9]. During thermochemical pretreatment, the macrofibrils have been observed to coalesce and form larger structures [10]. Even bundles as large as 140 nm have been reported [11] but harsh conditions can also result in much smaller fragments [12]. While the coalescence of fibrils and the increasing size in crystalline cellulose seem, however, diametral for efficient enzymatic hydrolysis, the non-cellulosic matrix polymers of lignin and hemicellulose add further complexity. In fact, neither pretreatment concept to date is fully understood for optimal enzymatic digestibility. To resolve the controversy between the different effects, changes have to be understood from the molecular structure via the polymer ultrastructure to the macrostructure of the tissue.

Ionic liquids (IL) pose an excellent substance for studying the effects of ions over a large concentration range. They can dissolve cellulose [13], and make lignocellulosic tissue swell [14]. Small amounts of water inhibit the dissolution of cellulose in IL [13] and molecularly dissolved cellulose recrystallizes into the crystalline lattice of cellulose II [15]. A treatment with ILs thus envisions the decrystallization of cellulose, thereby enhancing access to relevant chemical bonds.

An interesting pretreatment strategy was reported using EMIMAc at 115 °C that completely disintegrates the wood chips into single fibers within 1.5 h and gives approximately 65 wt% of sugars after 5 h of enzymatic hydrolysis [16]. This is much shorter than previously suggested and thus might offer an interesting mild, selective and quick pretreatment option for enzymatic hydrolysis. Nevertheless, anticipated prices of 2.5–50 $/kg [17] are clearly higher than molecular solvents and question an economic realization of process concepts with concentrated ionic liquid. Any water is thus appreciated from an economic point of view, in particular as biomass introduces water and the biotechnological downstream processes are universally based on aqueous systems. Hence, water has to be considered in these pretreatment concepts.

A few studies have already explored the molecular effects of EMIMAc in mixtures with water on wood. Shi et al. concluded that cellulose from switchgrass dissolves at 160 °C in the range of 50–80 wt% water content [18] but the relevance for mechanistic understanding is doubtful because such temperatures lead to degradation of both carbohydrates and the ionic liquid [19, 20]. Doherty et al. observed reduction of the cellulose crystallinity index in pretreated hardwood at less than approximately 10 wt% of water in EMIMAc [21]. No detailed study has yet addressed the effect of EMIMAc in the presence of water on wood.

The reports about the mechanisms of lignocellulosic pretreatment with pure IL are much more informative. Singh et al. demonstrated that lignin dissolution resulted in cell wall swelling followed by complete solubilization of the plant cell wall during ionic liquid pretreatment. Cheng et al. concluded an increased surface roughness as a result of removal of lignin and hemicellulose [22] but the enhancement of enzymatic saccharification rate was only related rather ambiguously to covalent bonds between lignin and carbohydrates. While no conversion of cellulose I into cellulose II was observed XRD revealed lattice expansion of the cellulose with pretreatment time. In contrast to the significant efforts in understanding aqueous-based biomass pretreatment, a multiscale analysis including mass balances, chemical analysis and structural investigations are missing for EMIMAc as the most interesting IL for pretreatment and its mixtures with water.

This study provides a systematic investigation of the effect of the EMIMAc ions with increasing concentrations in water on beech wood. This strategy enables to resolve the individual processes with varying concentration. First, we report the disintegration of wood chips on a macroscopic level using mass balances to determine the maximum acceptable water content for disintegration. Second, we investigate the process at the molecular level and structural scale to infer the mechanisms driving disintegration and facilitating enzymatic hydrolysis in aqueous mixtures of EMIMAc ions and water. This is carried out using a single set of pretreated material samples analyzed by compositional analysis, Raman spectroscopy, XRD, and SANS to account for the multi length scales of pretreatment. The results clarify the mechanism of pretreatment in ionic liquids and provide further insights for the design of solvent mixtures for biomass pretreatment processes.

## Results and discussion

### Effect of EMIMAc/water mixtures on beech wood chips

**Fig. 1:**
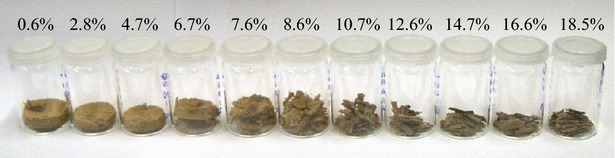
Morphology of beech chips pretreated in EMIMAc-water mixtures. The water mass fraction increases from *left to right* as indicated above the samples. While the material is completely disintegrated at low water content, it retains the original morphology at highest water content

Beech wood chips were exposed to EMIMAc mixed with water up to a water concentration of 18.5 wt% at 115 °C for 1.5 h. The morphology of the pretreated material (Fig. 1) is dramatically changed at low water concentrations. Essentially complete disintegration is achieved after pretreatment in concentrated EMIMAc (left-most sample in Fig. 1), consistent with previous observations [23]. The effect is observed up to a water content of 6.7 wt%. In samples treated with 0.6 and 2.8 wt% water in EMIMAc, beech wood fibers can be easily separated using a mortar and pestle. However, the sample at 4.7 wt% water in EMIMAc retains some structure that resists crushing by hand, and the sample at 6.7 wt% is not crushable. Careful analysis reveals that ray parenchyma cells persist in the wood that seems to retain the integrity of the material until being fully disintegrated (cf. scanning electron microscopy images in the Additional file 1).

At 7.6 wt%, many wood chips appear as regularly aligned wood fibers similar to that of native tissue. The proportion of intact tissue further increases in the sample with 8.6 wt% water, and the sample pretreated with 10.7 wt% water in EMIMAc exhibits no gross macroscopic disintegration at all. Only a few disintegrated fibers appear on the chip’s surface. No significant changes in the morphology in comparison to the original chips are observed in the chips treated with 12.6–18.5 wt% water in EMIMAc (Fig. 1). Hence, the transition between the fully disintegrated state and intact structure is identified between 7.6 and 10.7 wt% water in EMIMAc.

**Fig. 2:**
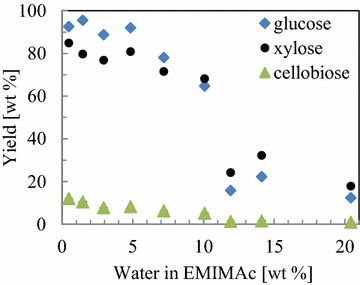
Sugars obtained after enzymatic hydrolysis of wood chips pretreated with varying water concentrations in EMIMAc (72 h, 45 °C using 1 ml buffer and 20 mg pretreated wood). Data points are the average of two hydrolysis experiments with deviations of ~1 wt%

The efficacy of pretreatment with EMIMAc in the presence of water is tested by measuring the hydrolyzed sugar contents of glucose, xylose and cellobiose by a commercial enzyme mixture. To give quantitative data on yields at small scale, the samples have been ground to a particle size <0.5 mm. The data in Fig. 2 shows that sugar concentration decreased sharply with increasing water content. Interestingly, all samples were hydrolyzed more efficiently in comparison to the untreated beech, which gives only 1 wt% of total carbohydrate yield (mainly glucose). The largest increase in sugar concentration relative to untreated wood is observed for glucose. More than 90 wt% yield based on cellulose is achieved with fully disintegrated material with up to 5 wt% water content in EMIMAc. Further increasing the water content causes these values to decrease and a large drop is observed with non-disintegrated samples treated with more than 10 wt% water. The data of cellobiose also exhibit a change at that concentration. While the hydrolysis of disintegrated samples results in small amounts of cellobiose, no cellobiose is detected in case of non-disintegrated material. Similarly, the xylose yield also shows such a transition upon disintegration. While the xylan in disintegrated material is hydrolyzed to an extent of about 80 wt%, and decreasing with the water content between 5 and 10 wt%, it is largely decreased above that water content reaching xylose yields between 20 and 30 wt%.

The results demonstrate the limited access for enzymatic hydrolysis in case of non-disintegrated material. The larger yield of xylose in comparison to glucose evidences a limited access to cellulose in the non-disintegrated samples. This is also reflected in the cellobiose yield. As the enzymatic activity of exo- and endoglucanases is higher compared to cellobiose-specific β-glucosidase in the commercial enzyme mixture [24], their activity is obviously sufficient to hydrolyze all the cellobiose from non-disintegrated wood.

The large increase in cellobiose and glucose concentration disintegrated state then identifies the benefit of pretreatment and disintegration. At water contents below the disintegration transition at 8.6 wt% the cellulose chains are increasingly available to enzymatic action, indicating that cellulose fibrils are fragmented more as water content decreases. In this state, the higher activity of exo- and endoglucanases exhibit the known behavior of limited cellobiohydrolase activity. Remarkably, the sum of cellobiose and glucose data give a quantitative yield of glucose from cellulose. The underlying molecular and structural changes of the composite structure of beech wood are to be investigated in the following sections.

### Mass balances of wood after EMIMAc/water pretreatment

**Fig. 3:**
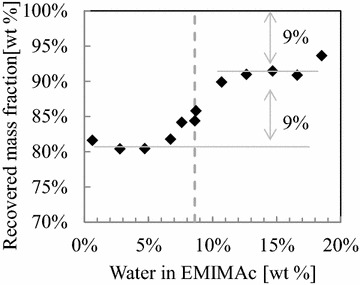
Recovered mass fraction after pretreatment of beech wood in EMIMAc/water mixtures. The *dashed line* indicates the transition center at 8.6 wt% water content in EMIMAc. The *numbers* depict the amount of extracted material at the two plateaus

Figure 3 shows the fraction of dry mass recovered after pretreatment. Above the disintegration transition, the mass recovery is ~91 % or higher, whereas below the transition it levels off at about 82 %. Between these two plateaus, a transition is observed with its center point at 8.6 wt%. The data indicate that significant amounts of wood components were extracted during the pretreatment, i.e., the extraction of 9 wt% at large water contents and the extraction of additional 9 wt% when wood chips are disintegrated. As the utilized beech wood consisted of polymers at more than 90 wt%, structural polymers seem to have been removed during pretreatment and washing.

This hypothesis was further tested by analyzing the composition of the solid samples by analytical acid hydrolysis and HPAEC-PAD (Table 1) according to a recently established protocol [25]. The data were computed as recovered mass relative to the initial dry mass of the native wood chips to present a mass balance of the pretreatment. In contrast to the established protocol, the correction for lost sugars using a sugar recovery standard was neglected because it resulted in larger masses of glucans in comparison to the native wood. This indicates that carbohydrates from pretreated wood possibly exhibit different degradation kinetics or pathways as compared to dissolved sugars in the sugar recovery standards.Other components tend to be unoriented

**Table 1:** Mass fractions of main components recovered in the solid samples after pretreatment

Water in IL	0.6	4.7	6.7	7.6	8.7	10.7	12.6	14.7	16.6	18.5	Native beech
AIL	12.5	14.2	11.9	14.7	15.5	13.8	14.3	14.8	14.7	11.4	22.1
ASL	2.7	2.5	3.0	3.8	2.6	2.5	2.7	2.4	2.6	2.6	
Glucans	38.5	33.9	40.4	40.6	44.0	44.8	40.3	41.8	44.0	42.8	42.6
Xylans	14.4	12.1	14.0	14.8	22.1	21.7	19.7	19.0	19.7	20.1	20.1
Galactans	1.3	1.0	0.7	1.3	1.3	1.1	0.9	1.2	0.8	1.2	2.8
Rhamnans	0.1	0.1	0.1	0.2	0.4	0.4	0.3	0.3	0.4	0.4	
Arabinans	0.4	0.4	0.5	0.5	0.6	0.6	0.4	0.4	0.6	0.5	0.8
Mannans	4.1	3.4	3.8	4.1	4.1	4.6	3.2	2.9	4.7	3.4	2.3
Total	71.3	65.1	71.3	76.1	88.1	87.0	79.2	80.3	84.9	79.7	91

Data are given in wt% calculated as recovered component mass per mass of native wood before pretreatment

*AIL* acid-insoluble (Klason) lignin, *ASL* acid-soluble lignin determined at 240 nm

Table 1 shows that Klason lignin or acid-insoluble lignin (AIL) content decreases from 22 wt% in native wood to approximately 14 wt% in the non-disintegrated samples and to 13 wt% when the material was disintegrated. Acid-soluble lignin (ASL) is determined as 2.7 wt% in all samples. The xylan content is slightly less than the native value of 20 wt% in the non-disintegrated wood, but decreases to approximately 14 wt% in the disintegrated state. Glucans show only a modest change with disintegration. Their fraction is close to the native value of 42.6 wt% in the non-disintegrated samples and decreases to 40 wt% for the disintegrated samples in concentrated EMIMAc.

The proportion of hemicellulose sugars mannose, galactose and arabinose retained after IL pretreatment appears less affected by the water content in EMIMAc (cf. Table 1). Mannans resist the pretreatment with EMIMAc/water and stay in the solid material. Similarly, galactans do not change over different water contents. In contrast, only half of the arabinans are found in all the samples and rhamnans are removed when disintegration is observed at 8.7 wt% water in EMIMAc and below. In total, compositional analysis shows 71 and 82 wt% on average for the disintegrated and non-disintegrated samples, respectively.

The chemical analysis of the solids thus shows only small changes with regard to the changing water content in EMIMAc and the disintegration. The most obvious change is the drop in xylan during disintegration between 7.6 and 8.7 wt% water in EMIMAc. Some arabinoxylan seems to be extracted from all samples, while the few galactoglucomannans likely stay in the wood during pretreatment. The drop in rhamnan concentration just when disintegration is observed is possibly related to removal of pectic substances by EMIMAc. A higher concentration of basic anions during pretreatment achieves a small reduction in Klason lignin, while glucans are retained to a large extend also in the disintegrated samples. The determined components meet the trend of the recovered masses in Fig. 3 with an unknown fraction of approximately 10 wt%.

A direct analysis of the pretreated material without wet chemical preparation was carried out using vibrational spectroscopy. All spectra show no evidence of imidazolium (cf. comparison of FT IR and FT Raman spectra with EMIMAc spectrum in Additional file 1: Figures S11, S12) confirming the efficacy of removing EMIMAc by washing. Furthermore, the absence of any signal at 1700–1780 cm^−1^, where acetyls and uronic acid groups in native wood contribute to vibrational spectra, indicates that hemicelluloses are deacetylated during the pretreatment.

**Fig. 4:**
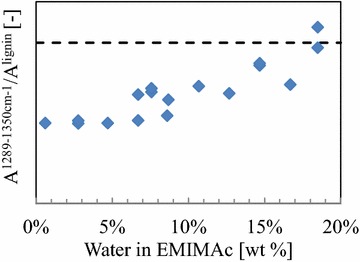
Semi-quantitative concentration of functional groups from Raman spectra. The areas were calculated to depict the relative change of functional groups The *dashed line* shows the average value obtained with native beech wood

Careful examination of spectra further reveals changes due to pretreatment, in particular at 1335/1314 cm^−1^. This group of vibrations was be assigned to a ring deformation mode with COC stretches of the two (O)CH3 groups in syringyl at 1331 cm^−1^ [26, 27] or aliphatic OH bending modes of hardwood lignin [28]. The integrated area of the range 1289–1350 cm^−1^ shows a decrease relative to the strong lignin vibrations at 1600 cm^−1^ with decreasing water content in EMIMAc (Fig. 4).

The spectroscopic data thus indicate that either aliphatic OH groups are cleaved at low water content below the point of disintegration or that EMIMAc selectively removes methoxy groups. The latter explanation would indicate that the syringyl to guaiacyl ratio is changed after pretreatment, which is roughly 3:2 in native beech [29]. Indeed, the cleavage of β-aryl ether bonds by EMIMAc has been observed [30–32] but it is commonly reported in combination with selective guaiacyl extraction [30, 33, 34]. Wen et al. observed a reduction in aliphatic OH in lignin that was recovered from EMIMAc pretreatment [34]. It might well be that aliphatic OH cleavage is involved in breaking the ether bonds. Furthermore, a closer look at the results of one of the other authors [33] reveals an initial decrease of the syringyl concentration at 1.5 h at 120 °C before guaiacyl extraction dominated in later stages. Hence, the Raman signal plausibly corresponds to a decrease in syringyl lignin that is extracted by cleavage of β-aryl ether linkages in the lignin. This result is also in line with the slightly lower Klason lignin content (Table 1) and demonstrates chemical lignin depolymerization during pretreatment in concentrated EMIMAc.

### Cellulose crystallinity due to pretreatment in EMIMAc/water mixtures

The structure of cellulose and its changes in response to IL treatments were then investigated by X-ray diffraction (XRD). The X-ray scattering arises from electron density distributions of all constituents of the samples. However, only cellulose gives rise to easily recognizable, oriented sharper diffractions. Other components tend to be unoriented in the samples and give rise to diffuse scattering that is generally treated as background beneath the relatively well defined scattering from cellulose. XRD enables investigation of cellulose crystallinity, molecular structure and assembly of cellulose fibrils, microfibril size and orientation and has been used extensively in analysing the effects of pretreatment of biomass and cellulose [12].

**Fig. 5:**
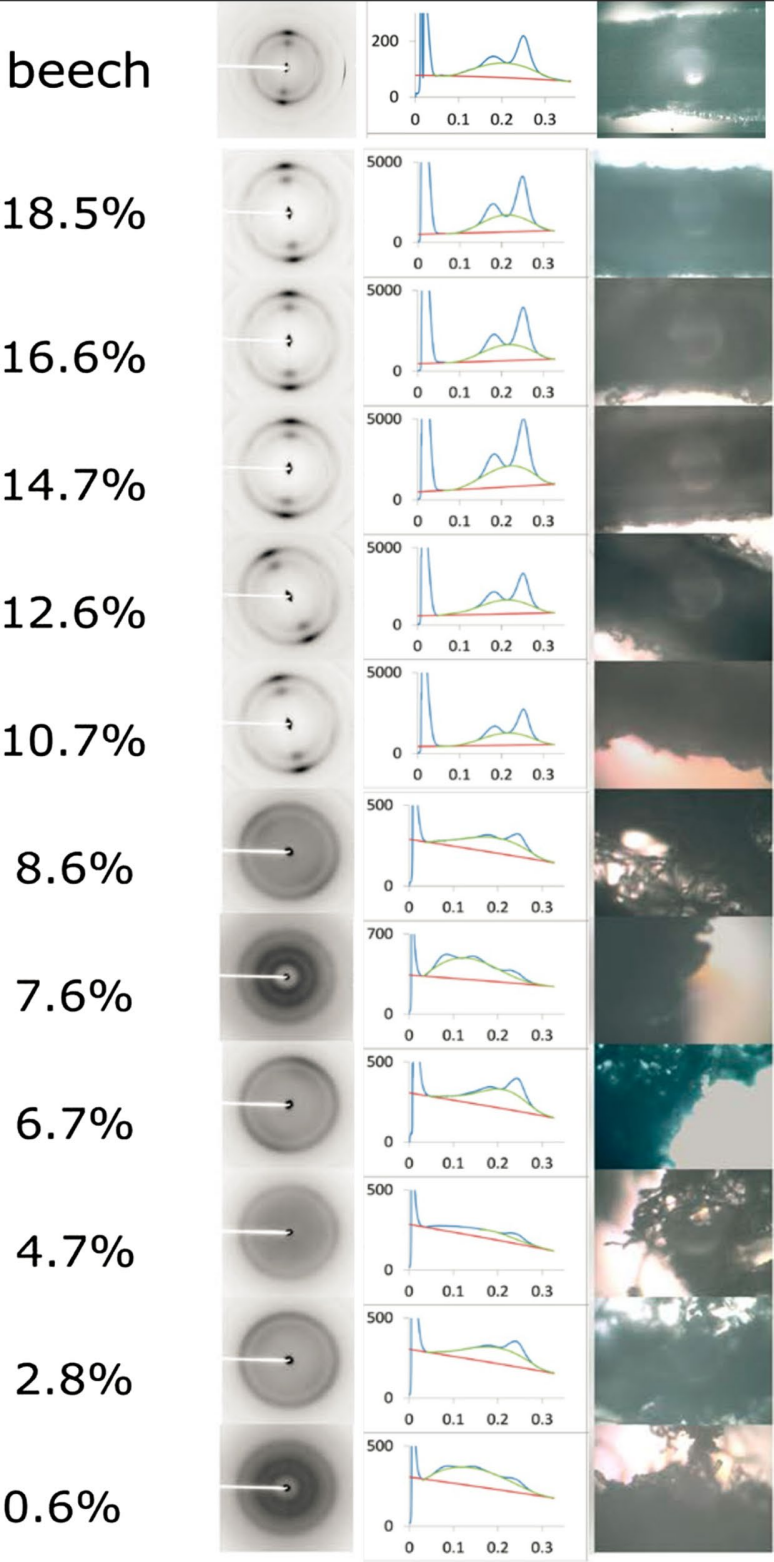
X-ray diffraction patterns (*left*), equatorial intensity distributions (*middle*), and light microscopic images of the regions of sample used for scattering experiments. The *fiber axis* is approximately *horizontal* in those patterns exhibiting orientation (i.e., with water content of 10.7 wt% and greater). The intensity distributions were derived by scanning in the radial direction across the two intense Bragg peaks and are plotted as a function of reciprocal coordinate *R* = 1/*d*, where *d* is the Bragg spacing [Å]

XRD patterns of native beech chips and pretreated material are shown in Fig. 5. Native beech exhibits all the characteristic reflections of Iβ cellulose. The cellulose fibrils are preferentially oriented in the wood chips of native material and those treated by EMIMAc/water mixtures at water contents of 10.7 wt% and more. At lower water concentrations, the fibrils are unoriented and give rise to circularly symmetric diffraction patterns.

The two strong equatorial reflections are indexed as (1–10)/(110) and (200) according to the unit cell of the cellulose Iβ structure (cf. [35, 36]). The latter reflection corresponds to the distance between stacked cellulose lattice planes, whereas the (1–10)/(110) reflections indicate the spacing of diagonal planes in the fibrils. The (1–10) and (110) reflections are superimposed in these patterns due to the symmetry of the orthorhombic unit cell [37]. The azimuthal distribution of intensity suggests well-oriented cellulose fibrils in the native beech wood, exhibiting only a single orientation and not exhibiting the double-orientation often associated with helical twisting of fibrils about plant cells [12, 38].

Samples pretreated with 10.7 wt% water content or greater exhibit x-ray patterns very similar to native material as can be seen in the patterns in Fig. 5. In this regime, background intensities decrease slightly and reflections are sharper in comparison to scattering from native material, consistent with removal of amorphous polymers as inferred from the compositional analysis summarized in Table 1. The observed Bragg peaks shift to slightly larger scattering angles (reflecting a slight tightening of the packing of cellulose molecules within the fibrils). However, patterns from samples treated with IL diluted with 8.6 wt% or less water reflect dramatic changes in molecular architecture. The patterns show essentially no orientation and broader, weaker reflections on a baseline of amorphous scattering that decreases rapidly with increasing scattering angle, but no reflections attributable to the thermodynamically more stable cellulose II polymorph are detected. It can be concluded that EMIMAc with water at concentrations less than 8.6 wt% leads to dissolution of the cellulose in wood without recrystallization upon regeneration of the pretreated wood from the ionic mixture.

Interestingly, the samples treated with water contents of 0.6 and 7.6 wt% (Fig. 5) exhibit a strong, somewhat diffuse reflection at a spacing 0.08 Å^−1^ (corresponding to a structural periodicity of 12 Å) which is not accounted for by the Iβ crystalline lattice of cellulose. Samayam et al. also observed a similar reflection at 11.5 Å and suggested a face-to-face stacking of glucose rings in the cellulose with IL ions in between causing the much larger structures [15]. However, due to the very low concentration of IL—spectroscopic methods could not detect any—this effect will not be considered further in this study.

**Fig. 6:**
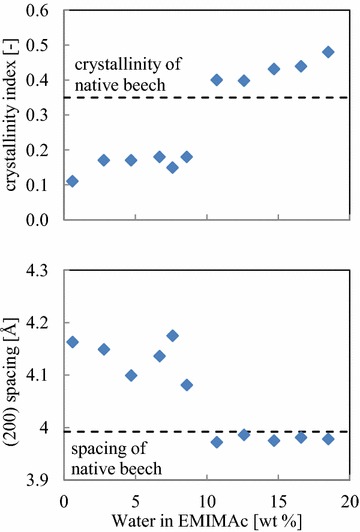
Crystallinity index (*top*) and spacing of the (200) diffraction of the samples pretreated in EMIMAc/water mixtures. Both the crystallinity and the spacing change when the material is disintegrated

The structure of crystalline cellulose in these samples is further analyzed based on these reflections in terms of crystallinity index and lattice distances (Fig. 6). The cellulose crystallinity index is the percentage of cellulose in a crystalline form (the remainder being structurally amorphous) and is derived from the equatorial intensity distribution. At a water content of 10.7 wt% or more, the crystallinity index is greater than that observed for native material, consistent with the observation of sharper reflections (cf. Fig. 5). At water contents below that of the disintegration transition, the observed crystallinity plummets, becoming less than half that of native and indicating a profound disruption of cellulosic fibrils.

The spacing between sheets of cellulose molecules in the fibrils of cellulose Iβ is inversely proportional to the position of the (200) reflection. In native material, the (200) spacing corresponds to an inter-sheet distance of 3.99 Å. Treatment with IL diluted by more than 8.6 wt% water does not alter this length significantly (Fig. 6), but it increases to ~4.18 Å in samples treated with lower water contents. This difference is significant, since the sample-to-sample deviation of the measurement is much smaller and reflects a re-packing of cellulose molecules in the fibrils that remain intact after treatment.

In conclusion these results demonstrate that the crystalline cellulose in beech wood is efficiently broken down into mostly amorphous cellulose by EMIMAc with a water content of 8.6 wt% or less. The data depict cellulose in the treated material as disoriented, disordered and disrupted at a molecular level with only a small contribution of the residual crystalline cellulose Iβ, but not cellulose II. In fact, the swelling and decrystallization of cellulose fibrils takes place over the same range of water content where macroscopic disintegration is observed.

### Structural changes of the cell wall by EMIMAc/water mixture

Samples from the set in Fig. 1 have been analyzed with small-angle neutron scattering (SANS) to elucidate their nanostructure. Before measurement, the dry samples were soaked in D_2_O thoroughly to increase the contrast between the hydrophilic voids and the hydrogen containing constituents of cellulose, hemicellulose and lignin in the cell wall. This preparation resembles a rewetting step after drying (which was applied in this study for mass balancing) to reveal the structure of the pretreated material under the conditions of enzymatic hydrolysis.

The scattering patterns (Additional file 1: Figure S13) reveal similar trends to those observed by XRD. Namely, the scattering from native wood exhibits a preferential orientation of cellulose fibrils. The patterns from material treated with relatively high water content of 12.6 and 8.6 wt% in the IL continue to exhibit a preferential orientation, but show a change in the scattering intensity distribution. While the anisotropy is less pronounced at 8.6 wt%, no orientation in scattering is observed after the treatment in 7.6 or less water in EMIMAc. The absence of distinct meridional features points out that there is no larger periodic structure in fiber direction until the material is disintegrated into isotropic material. The following analysis is therefore performed for the equatorial intensity distribution only.

**Fig. 7:**
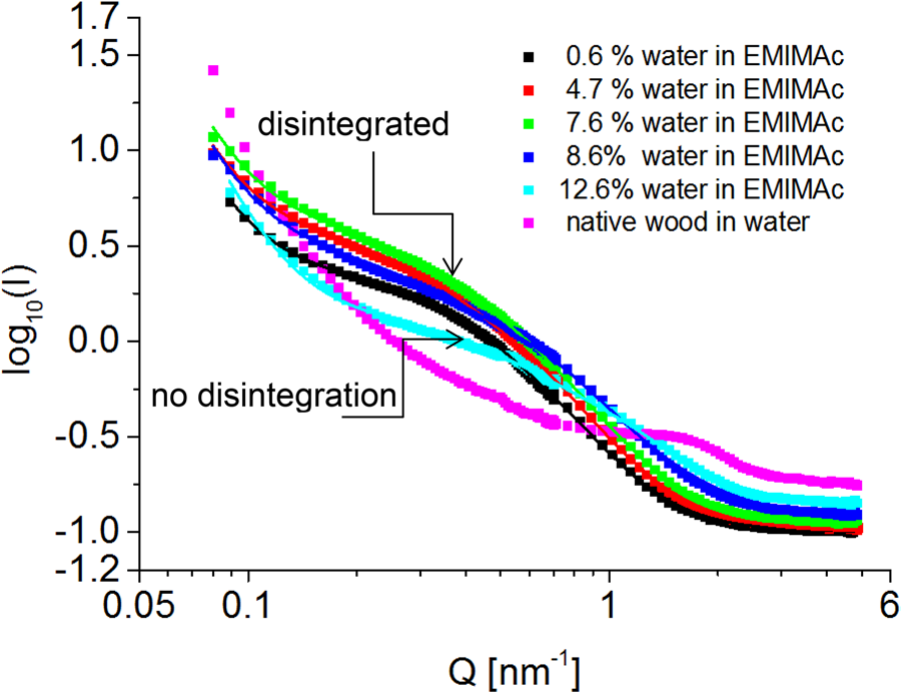
Radially averaged SANS profiles of native and pretreated wood samples in D_2_O. While native wood shows a characteristic nanostructure of the cell wall, the data indicate the formation of larger structures due to pretreatment in EMIMAc/water

The SANS scans in Fig. 7 show a distinct equatorial diffraction feature (at *Q* ≈ 1.6 nm^−1^) in native wood. This intensity maximum can be attributed to the regular lateral packing of crystalline microfibrils in the direction normal to the fiber axis (cf. [39]). Calculation of the lateral dimension in the samples gives a characteristic dimension of $d = \frac{2\pi }{{Q_{ \hbox{max} } }} =$ 3.9 nm, which is in good agreement with the finding of a center-to-center distance of 4 nm between the microfibrils [3, 39, 40].

When EMIMAc or mixtures with water are used for pretreatment of the wood, the peak at 1.6 nm^−1^ disappears and larger structures are formed as indicated by the higher intensities at lower *Q*. The data from non-disintegrated material (12.6 wt% water in EMIMAc) show a scattering shoulder at *Q* = 0.6 nm^−1^. With progressive disintegration in the more concentrated samples, the signals of disintegrated material show a shoulder at *Q* = 0.3 nm^−1^ that indicated a further increase of the lateral dimension in comparison to the sample pretreated with 12.6 wt% water in EMIMAc.

A combined evaluation of XRD and SANS data enables the assignment of these results to structural features of the wood. XRD shows a distance of 0.39 nm between the cellulose chains across the (200) crystal lattice in the native wood. According to recent investigations on hardwood, a microfibril bundle consists most likely of 8 sheets [40], which corresponds to a microfibril diameter of 3.12 nm based on the (200) spacing in our case. The repeating distance from SANS of approximately 3.9 nm is thus considerably larger than the microfibril diameter estimated from XRD measurements and provides a measure of the mean distance between microfibrils in these samples.

Therefore, the different characteristic lengths from XRD and SANS hint at the structure in the native cell wall of wood. The SANS contrast originates from deuterium. If the crystalline microfibril was deuterated, then the crystalline cellulose would have shown a broadened (200) distance, which is not observed in XRD. Disordered cellulose surface chains of the fibril are much more accessible for deuteration [41] than crystalline cellulose, in particular if the hydrophobicity of the (200) lattice planes of cellulose is considered [40]. The crystalline core of microfibrils is thus surrounded by D_2_O and or non-crystalline molecules in the native state (cf. [42]). The microfibrils are thus separated by hydrophilic moieties that are easily deuterated and attract interfibrillar water.

The crystalline features of the sample of 12.6 wt% water do not change considerably in XRD although the patterns are sharper and the crystal size [the width of the (200) peak] is slightly increased in comparison to native wood. Hence, the crystalline core of the microfibrils does not seem to be altered at that water concentration. However, the peak at *Q* = 1.6 nm^−1^ is not present in pretreated samples and a new scattering shoulder at *Q* = 0.6 nm^−1^ suggests a closer packing in the sample with 12.6 wt% water in EMIMAc. Without knowing the precise structure, we estimated the radius of gyration (*R*_g_) from the SANS data by model-based analysis of the curves with fewest possible assumptions (cf. “Small-angle neutron scattering” for details).

**Fig. 8:**
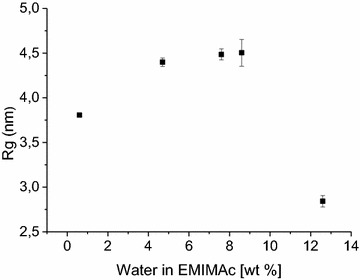
Radii of gyration (*R*_g_) of cellulose fibrils as a function of the water content of EMIMAc during the disintegration process. The radii are larger in the disintegrated samples than those in the sample at 12.6 wt% water content during pretreatment and seem to decrease after processing in concentrated EMIMAc

Figure 8 displays the *R*_g_ of the analyzed samples. The data point at 12.6 wt% content relates to a *R*_g_ ≈ 2.8 nm. The crystalline bundles thus exhibit a diameter of approximately 5.6 nm in the sample treated with 12.6 wt% water in EMIMAc. As XRD reveals an inter-sheet distance of approximately 0.399 nm in this sample, microfibrils seem to have aggregated in a group of at least four former microfibrils. Fully disintegrated samples treated with a water content of 8.6 wt% and less show rather large radii of gyration of ca. 4.5 nm. In concentrated EMIMAc, the *R*_g_ is reduced to 3.85 nm. Hence, the disintegration leads to isotropic aggregates on the scale of 9 nm, which reduces to approximately 7.7 nm as a result of the pretreatment in concentrated EMIMAc. As most of the fibrils have been converted into amorphous structures, no crystal feature can be assigned to these structures.

### Discussion of the pretreatment mechanisms in EMIMAc/water mixtures

The combination of chemical component analysis and structural investigation sheds light on the pretreatment mechanisms in EMIMAc. As the mere removal of hemicellulose would create a void of less than one nm, insufficient for large cellulases (~5 nm, [8]), the process is clearly more sophisticated. Hence, the key question is how the mixtures of EMIMAc/water result in more efficient access for enzymes to the cellulose.

On the molecular scale, the strongly ionic conditions of EMIMAc/water mixtures comprise highly basic anions that result in deacetylation (cf. “Mass balances of wood after EMIMAc/water pretreatment”; Additional file 1: Figure S11, S12). It was already reported in EMIMAc at 120 °C [30] and also at temperatures as low as 60 °C [43], which suggests deacetylation being not limited by kinetics (temperature or time) in these mixtures. The effect of deacetylation on enzymatic hydrolysis is, however, not that clear. It plausibly provides loosening of the carbohydrate structure. There are, however, positive [43] and negative [44] correlations of deacetylation with enzymatic digestibility. The enhanced digestibility may be due to enhanced accessibility of the xylan itself for hydrolysis which then in turn enables better access to cellulose in comparison to the native wood (cf. [45] and sugar yields in Fig. 2). A tailored mixture of enzymes for hydrolysis of C6 and C5 polymers would possibly enhance deconstruction of pretreated biomass.

The detection of amorphous and/or crystalline cellulose (Fig. 6) motivates mechanistic differentiation between the regimes of disintegration and non-disintegration at different EMIMAc/water mixtures (cf. samples in Fig. 1). The effects in the non-disintegrating regime seem to be related to water between microfibrils and removal or reorganization of polymers. In fact, water constrained by non-cellulosic polymers in the vicinity of cellulose was evidenced to play an important role in enzyme binding to the cellulose [46]. The authors showed that water-insoluble hemicelluloses were even more inhibitory to enzymatic hydrolysis of cellulose than lignin, which implies that the removal of these components or the constrained water therein from the cellulose might improve the enzymatic hydrolysis. In this line, the SANS contrast at *Q* ≈ 1.6 nm^−1^ in this study (Fig. 7) was previously monitored during enzymatic hydrolysis of microcrystalline cellulose [47] and revealed enhanced hydrolysis rate upon disappearance of this peak. Consequently, expelling water from the microfibrils is regarded a common mechanism of other aqueous pretreatment processes [3]. Although the removal of water could be either a consequence or effect of the extraction of hydrophilic carbohydrates from the interfibrillar surfaces, both result in the observed coalescence of microfibrils as means of aqueous pretreatment.

Indeed, the compositional analysis (Table 1) suggests some extraction of structural components, i.e., arabinans, lignin and a few xylans. The latter are mainly located in the secondary wall [48] and assigned to xyloglucan which is likely due to the interfibrillar water between microfibrils [40]. The removal has been observed before at temperatures above 140 °C as in Kraft pulping with coniferous wood [49] or other pretreatments [3] and Froschauer et al. [50] achieved selective xylan extraction in EMIMAc/water mixtures between 15 and 20 wt% water at 60 °C within 3 h. Hence, the results of this study identify dilute EMIMAc/water mixtures to be an effective pretreatment medium at mild temperatures.

At first sight, the hypothesis of coalesced fibers as determined by SANS (Fig. 8) seems diametral for enzymatic hydrolysis as this is usually less beneficial for accessibility [10], and a considerable decrease in crystallinity is not achieved either. However, the enzymatic hydrolysis is not always negatively correlated with the crystallinity of wood celluloses [51] but depends on preferential binding to the hydrophobic face of the cellulose microfibrils [52]. Hemicelluloses and pectins that cover the hydrophobic surface of cellulose between microfibrils [53] will thus decrease enzymatic activity. Hence, removal of these hydrophilic substances from cellulose fibrils is key for enzymatic hydrolysis when the cellulose structure is not altered.

In concentrated EMIMAc/water mixtures, the disintegration eventually destroys cell wall integrity. It is critically determined by lignin and hemicellulose in the middle lamella and the large secondary wall. Rhamnose was observed to be extracted completely in this study upon disintegration (Table 1). The rhamnans and also arabinans likely originate from pectins, which are responsible for cell wall adhesion in the middle lamella [54].

The swelling is due to expansion of microfibrils in the secondary wall, which are tethered by pectins and in particular xyloglucans in native wood [48, 53, 55–57]. Upon removal of these substances and in particular xyloglucan, which is intercalated between microfibrils and plays a role in the lignin-carbohydrate structure [2], the microfibril swells enhanced by the space required for the additional ions (cf. [10]).

The lignin network, however, limits the flexibility of the cell wall. Weakening of the lignin can be of physical nature as the temperature in the set of experiments is close to the melting temperature of lignin at around 120 °C [4]. Furthermore, the results showed that lignin is also dissolved during pretreatment. The extraction of lignin by EMIMAc was found to take place mainly in the secondary wall [58]. It is the syringyl lignin, which is most abundant in the secondary wall [59] and which is easier to extract and more reactive [60]. Indeed, Raman spectroscopy suggests a reduction of phenolic side chains (cf. Fig. 4), which hints towards the extraction of syringyl lignin. The slight reduction of lignin in the disintegrated samples indicates a slightly enhanced extraction of lignin in comparison to the aqueous regime, which might be due to reactive cleavage of aliphatic bonds in concentrated EMIMAc.

The ionic liquid eventually leads to dissolution of cellulose. The dissolution results in separation of the sheets of cellulose by the EMIMAc that localizes between them and increases the intersheet distances in the cellulose. Macroscopically, this leads to swelling until disintegration of the lignin depleted structure. Only small fractions of the microfibrils remain crystalline after pretreatment, which could be due to incomplete dissolution. Hence, we hypothesize that the action of delignification and swelling starts from the lumen of the cell and eventually disintegrates the tissue, making the pectic substances of the middle lamella much more accessible.

However, the cellulose is not undergoing bulk recrystallization into cellulose II as observed with pure cellulose, but results in amorphous cellulose embedded in the cell wall. Wu et al. [61] studied a combination of dissolution and delignification and concluded that lignin precludes the recrystallization of cellulose. It is also known that macrofibrils are very robust even in case of complete disintegration of the cell wall [10]. Their dimension with a diameter of 14–23 nm [9, 37, 42] is larger than the aggregates observed in this study with SANS. It is thus likely that the remaining structure of the former macrofibrils in the disintegrated cell wall contains the cellulose and freezes their amorphous state when EMIMAc with it hydrophobic moieties is removed during washing. The resulting fully amorphous cellulose offers much more hydrophobic surfaces in a much more porous scaffolding. This combination of multi length-scale processes thus provides easier access for enzymatic hydrolysis.

## Conclusions

The pretreatment with 1-ethyl-3-methylimidazolium acetate (EMIMAc)/water mixtures was analyzed systematically up to 18.5 wt% water content on several length scales to elucidate the beneficial mechanisms for enzymatic hydrolysis. The macroscopic analysis shows the wood chips to be disintegrated by EMIMAc with water contents up to 8.6 wt%, which coincides with the equimolar composition of IL to water. However, all EMIMAc/water mixtures result in an improved enzymatic hydrolysis. The combined application of compositional analysis, spectroscopy as well as scattering techniques of XRD and SANS revealed the following mechanisms:

A first step for improved hydrolysability is the removal of some structural polymers and acetyl groups. Water between microfibrils is expelled indicating removal of hydrophilic matrix polymers from the cellulose microfibrils and likely allows for coalescence of the uncovered microfibrils. Aqueous mixtures with EMIMAc thus resemble established aqueous pretreatment. Concentrated EMIMAc then achieves dissolution of xyloglucan, defunctionalization of lignin, and dissolution of the cellulose, which results in extensive swelling of the microfibrils in the secondary wall. The amorphous state of cellulose in the remaining cell wall structure remains even when the solvating ions are removed. Afterwards, the material is much better accessible as shown by wet chemical analysis and enzymatic hydrolysis. These mechanisms should be exploited for the design of mild temperature pretreatment processes using ionic mixtures at controlled concentration.

## Methods

### Materials

Wood chips of 10 mm × 2 mm × 0.7 mm were made from beech (*Fagussylvatica*) veneer (Kohl GmbH, Cologne, Germany) with the fiber axis in longitudinal direction. The moisture content of the wood chips was analyzed by drying at 105 °C overnight. The mass fractions of lignin (22.1 wt%), glucans (42.6 wt%), xylans (20.1 wt%), galactans/rhamnans (2.79 wt%), arabinans (0.76 wt%), mannans (2.30 wt%), ash (0.3 wt%), and extractives (2.6 wt%) were determined using an established analytical protocol [62–64] scaled-down to a sample mass of 150 mg. The data are reported in polymeric mass fractions to account for the hydrolysis and for the unknown structural origin of the determined monosaccharides (i.e., glucan instead of cellulose).

EMIMAc (>95 wt% purity) was obtained from Iolitec (Heilbronn, Germany). The water content of the IL was determined using Karl-Fischer titration (Coulometer 831, Metrohm, Filderstadt, Germany). Acetone was p.a. (BDR Prolabo), and deionized water (conductivity < 10 µS) was used throughout.

### Wood pretreatment in EMIMAc/water mixtures

The procedure follows the strategy applied by Viell et al. (2011) and Viell et al. (2013). First, a mass of 0.5 g of wood chips was added to a centrifugal tube (50 ml). Second, approximately 7.8–9.5 g of EMIMAc was weighed into the tube and the corresponding amount of water was added subsequently to obtain a total sample mass of 10 g. A magnetic stirring bar (Komet 15) was added before closing the centrifugal tube, which was then placed in a hot heating block that also enables magnetic stirring. This marked the starting point of the pretreatment at 115 °C for 1.5 h.

After the pretreatment time, the samples were cooled down, analyzed by infrared spectroscopy (see below), and the dark viscous mass was washed 7 times with water and acetone (1:1 v/v) to remove the IL and any dissolved material effectively. The washing was carried out in weighed filter crucibles (POR 4, ROBU Hattert, Germany), which determined the dry mass of pretreated wood after drying overnight at 105 °C.

The total mass of water in the samples was hence determined by the water in the wood (moisture content: 0.064 g/g_dry wood_), the water content in the IL (0.3 wt%), and the added water to the particular experiment. The sum of these values gives the total water content. A separate analysis verified that the calculated, total water content of the pretreatment samples did not differ from the liquid water content after the experiment. FT IR spectroscopy was utilized to analyze the liquid, which was evaluated by a non-linear model [65] to calculate the water content. The data show a good correlation between measured and calculated values (Additional file 1: Figure S14) with a deviation of ±2.3 wt%, which is within the range of the error of the utilized model for spectral quantification. Hence, the total calculated water content in EMIMAc can be taken as physical means to characterize the effect of water in the IL on the disintegration of wood during pretreatment.

### Enzymatic hydrolysis

The enzymatic hydrolysis procedure was performed by modifying the protocol in Engel et al. (2012). 20 mg pretreated beech wood in 1 ml 0.1 M sodium acetate buffer pH 4.8 was hydrolyzed using 580 μl Celluclast 1.5 l/g wood (Celluclast 1.5L ATCC 26921 is from Novozyme, Denmark). The protein content was 84 mg protein/g biomass, and the according FPU was 19.8 FPU/g biomass. The mixture in 2 ml Eppendorf tubes was incubated at 45 °C and 900 rpm in a thermo mixer MHR 23 (HLC Biotech, Bovenden, Germany). The reaction was stopped by incubation of the samples at 100 °C for 10 min. After centrifugation for 10 min at 10,000×*g*, the supernatant was diluted in a proper ratio and filtered through 0.2 µm PVDF filter. The sugar concentrations in the supernatant were determined by high-performance anion exchange chromatography (cf. “Chromatographic carbohydrate analysis”).

### Chromatographic carbohydrate analysis

Monosaccharides originating form two-step acid hydrolysis (cf. [64]) of pretreated wood were determined using high-performance anion exchange chromatography coupled with pulsed amperometric detection (ICS-5000+, ThermoScientific, USA). This system was equipped with a CarboPacTM PA100-column for monosaccharide separation (ThermoScientific, USA). The flow was set to 1 mL/min. The column temperature was 40 °C and the gradient established according to Anders et al. [25].

In contrast to the hydrolyzates from two-step acid hydrolysis, the enzymatic hydrolyzates allow for a shorter method as the number of expected products is limited due to a much more specific breakage of cellulose and hemicellulose bonds. In this context the method described by Anders et al. [25] was shortened to 11 min as only 100 mM NaOH (A) and 100 mM NaOH/500 mM NaOAc (B) were used. The gradient is shown in Table 2.

**Table 2:** Elution profile for the sugar determination in enzymatic hydrolyzates using HPAEC-PAD

Time (min)	% NaOH	% NaOH/NaAc
−5	99	1
0	99	1
6	75	25
8.5	30	70
9	99	1
11	99	1

### Optical spectroscopy

The liquid mixtures were analyzed by spectroscopy to determine the water concentration after the experiment and gain insight into the change in composition after pretreatment. Fourier transform infrared (FT IR) spectroscopy equipped with a fiber-optical probe (Tensor 27, Bruker/Dr. Bastian GmbH, Ettlingen/Wuppertal, Germany) was used to check the composition of the EMIMAc/water mixtures. The analysis comprised of 100 spectra during acquisition (4 cm^−1^ resolution) with OPUS 6.5 (Bruker, Ettlingen, Germany) and subsequent spectra evaluation. The latter was carried out with an established non-linear multivariate method (Viell and Marquardt [65]).

The solids after drying were also analyzed spectroscopically. An FT Raman spectrometer (Bruker MultiRAM) was used with an excitation laser at 1064 nm in backscattering geometry. The solid samples were scanned a 100 times with 250 mW. Solid state FT IR spectra were acquired with the instrument described above by pressing the samples firmly against the ATR diamond of the fiber optical probe.

The spectra were semi-quantitatively evaluated using indirect hard modeling [66]. The modeling was carried out with Peaxact (version 3.6.3, S-Pact, Aachen, Germany). The area of distinctive peaks was computed by integrating between 1530 and 1708 cm^−1^ for lignin and 1289 and 1350 cm^−1^ using a linear baseline.

### X-ray diffraction

X-ray experiments were carried out at the Advanced Photon Source (APS) at Argonne National Laboratory, beamline 23ID-D (GM/CA) using a beam diameter of 5 μ for a wide angle X-ray scattering (WAXS) in the scattering range of 2.3–100 Å. The system consisted of a mini-beam collimator, a magnetically indexed kinematic mount and a precision motion system. Excellent scatter protection was achieved by nesting of the pinhole. The pinholes were held into the uni-body with a cap that also served as a back scatter guard. Accurate positioning of the sample was achieved by use of an on-axis microscope system accommodating axial holes in the optical elements such that the X-ray beam traveled down the optical axis of the microscope. Patterns were recorded using a MARmosaic300 detector having 4096 × 4096 pixels with a pixel size of 73.24 μm. The specimen-to-detector distance was 300 mm and the X-ray wavelength was *λ* = 1.033 Å. The sample was oscillated through 1° during the 10 s exposure. Five patterns were collected from each sample to check for heterogeneity.

The intensity distribution is presented in reciprocal coordinates according to $R = 1/d = 2\sin \theta /\lambda$ where *d* is Bragg spacing, 2*θ* is scattering angle, and $\lambda$ is wavelength.

The integral width in reciprocal coordinate *R* is defined as the peak area divided by the peak height for a reflection of Bragg spacing *d*, and this gives the inverse of coherence length [12].

The cellulose crystallinity index was defined as the crystalline cellulose content in the total cellulose which was composed of crystalline and amorphous cellulose components. Assuming the weight of the exposed object by X-ray was proportional to the integrated scattered intensity, the cellulose crystallinity index from the X-ray diffraction was defined according to

1 $$c_{\text{index}} = \frac{{I_{{{\text{crystalline}}\,\,{\text{cellulose}}}} }}{{I_{\text{cellulose}} }} = \frac{{I_{{{\text{crystalline}}\,\,{\text{cellulose}}}} }}{{I_{{{\text{crystalline}}\,\,{\text{cellulose}}}} + I_{{{\text{amorphous}}\,\,{\text{cellulose}}}} }} = \frac{{I_{tot} - I_{bk2} }}{{I_{tot} - I_{bk1} }} = \frac{{c_{{{\text{crystalline}}\,\,{\text{cellulose}}}} }}{{c_{\text{cellulose}} }}$$

where *I*_*tot*_ was the integrated intensity (blue curve in Fig. 5). *I*_*bk*1_ was the integrated intensity of the baseline (red curve in Fig. 5), and *I*_*bk*2_ was the integrated intensity of the background (green curve in Fig. 5). The cellulose content in biomass was written as

2 $$c_{\text{cellulose}} = \frac{{I_{\text{cellulose}} }}{{I_{tot} }} = \frac{{I_{{{\text{crystalline}}\,\,{\text{cellulose}}}} + I_{{{\text{amorphous}}\,\,{\text{cellulose}}}} }}{{I_{\text{cellulose}} + I_{\text{lignin}} + I_{\text{hemicellulose}} + I_{\text{others}} }} = 1 - \frac{{I_{bk1} }}{{I_{tot} }}.$$

The crystalline cellulose content in biomass was therefore determined according to

3 $$c_{{{\text{crystalline}}\,\,{\text{cellulose}}}} = \frac{{I_{\text{cellulose}} }}{{I_{tot} }} = 1 - \frac{{I_{bk2} }}{{I_{tot} }},$$

where

4 $$I_{bk2} = I_{{{\text{amorphous}}\,\,{\text{cellulose}}}} + I_{bk1} .$$

Equation (4) indicates that the amorphous cellulose scattering (giving the broad maximum at about 5 Å in Bragg spacing) is derived from the difference between the two background curves of *I*_*bk*1_ and *I*_*bk*2_. If the non-cellulose components in the sample, of which scattering curves have not yet been fully characterized, contribute to the broad maximum, the cellulose crystallinity index measured in this study may be underestimated, but is still valid for a relative comparison.

### Small-angle neutron scattering

SANS experiments were carried out at the KWS-2 diffractometer in operation at the research reactor FRM-II with the host organization Heinz Maier-Leibnitz Zentrum (MLZ) in Garching, Germany [KWS2]. The wavelength of the neutrons was 4.8 Å. The collimation was set to 8 m with the corresponding sample to detector distances of 8 and 1 m for the *Q*-range of interest. The scattering vector *Q* is given by *Q* = (4*π*/*λ*) sin (*θ*/2). The intensity *I* (*Q*) is collected on an area detector as a function of the scattering angle θ, or the scattering vector *Q*, respectively. The experiments were carried out at room temperature. So called sandwich-cells with quartz windows were used as sample holders. All scattering intensities are on absolute scale by calibration with a secondary Plexiglas standard. The use of heavy water (D_2_O) provides a natural contrast between the soaked cavities inside the wood and the solid materials, mainly the cell walls including cellulose, hemicellulose and lignin that contain natural hydrogen. Since we stay at semi-quantitative modeling of the absolute intensities (in cm^−1^) we did not go in detail with exact scattering length densities of all materials in the wood. However, the* Q*-scale is absolute and allows for measuring absolute sizes, especially the cellulose fibril structure.

Model-based analysis of the SANS curves in Fig. 8 using the following function

5 $$I\,(Q) = A_{1} Q^{{ - P_{1} }} + A_{2} \left( {\exp ( - Q^{2} R_{g}^{2} /3 + \frac{{P_{2} }}{{\varGamma (P_{2} )}}\left( {\frac{{{\text{erf}}^{ 3} (kQR_{g} /\sqrt 6 )}}{{QR_{g} }}} \right)^{{P_{2} }} } \right) + B_{{ck{\text{grd}}}} ,$$

with the amplitudes *A*_1_ and *A*_2_, and the exponent *P*_1_ ≈ 4 describing the Porod scattering from the cell walls against the D_2_O filled cells; with the amplitude *A*_2_, the radius of gyration *R*_g_ and the exponent *P*_2_ ≈ 3 describing the whole coalesced fibril scattering (empirically *k* = 1.06, cf. Beaucage [67], and applied in [42]); and with *B*_ckgrd_ being the incoherent background from mainly hydrogen atoms in the scattering volume. At the present stage, the modeling assumes globular structures that we see in the intermediate *Q*-range (0.2–1 nm^−1^) because the typical scattering of cylinders would nearly be invisible from the strong Porod scattering at lower *Q*. In this sense, the Guinier scattering describes the structure in the two equatorial dimensions, and details about the length are hidden experimentally. Fitting Eq. (5) to the experimental data enables estimation of the parameter *R*_*g*_ as quantitative means of the scattering structures in the lower *Q* region. The radius of gyration *R*_g_ relates to the diameter *R*_c_ of a cylindrical cross-section by *R*_*g*_ ≈ *R*_*c*_.

## Additional file

10.1186/s13068-015-0422-9 Supplementary images and diagrams from the set of samples, i.e., SEM images of disintegrated wood, spectral data of FTIR, RAMAN and SANS.

## Abbreviations

FT IR: Fourier transform infrared
FT Raman: Fourier transform Raman
WAXS: wide-angle X-ray spectroscopy
XRD: X-ray diffraction
SANS: small-angle neutron scattering
EMIMAc: 1-ethyl-3-methylimidazolium acetate
IL: ionic liquid
HPAEC-PAD: high-performance anion exchange chromatography-pulsed amperometric detection
UV/Vis: ultraviolet/visible spectroscopy
