# Supplementary material for: Multi-scale processes of beech wood disintegration and pretreatment with 1-ethyl-3-methylimidazolium acetate/water mixtures
Source: Biotechnol Biofuels. 2016 Jan 8;9:7. doi: 10.1186/s13068-015-0422-9 (PMC4706671; doi:10.1186/s13068-015-0422-9)
Supplement: Supplementary file 1 — 10.1186/s13068-015-0422-9 Supplementary images and diagrams from the set of samples, i.e., SEM images of disintegrated wood, spectral data of FTIR, RAMAN and SANS. [file 13068_2015_422_MOESM1_ESM.docx]

# Additional File 1

Figure 10. Different states of disintegration at the specified water contents in EMIMAc. While the cells are completely disintegrated at low water content but intact (top), ray parenchyma cells are not disintegrated at increasing water content (magnified).

Figure 11. FT IR spectra of dry disintegrated wood from the experiments with 0.6, 2.8, 7.6 and 8.6 wt % water content (top) and the spectrum of EMIMAc (bottom) with the strong asymmetric (as) and symmetric vibrations (ss) of carboxyl and imidazolium ring. The comparison of both spectra in particular at the indicated absorptions of EMIMAc gives no evidence of substantial residual EMIMAc in the solid material.

Figure 12. FT Raman spectra of disintegrated wood from the experiments from 0.6 up to 8.6 wt % water content (top) and the spectrum of EMIMAc (bottom). The comparison of both spectra in particular at the strongly scattering molecular vibrations of EMIMAc gives no evidence of substantial residual EMIMAc in the analyzed solid material. Considering the depth of penetration of ~268 µm of the Raman analysis (determined in supplementary experiments with cellulose filter paper), this confirms the efficacy of washing.

native beech chip:

Figure 13. SANS diffraction patterns of samples soaked in D_2_O (fiber axis horizontal in case of native beech). While the pretreated samples and its patterns are shown on top, the patterns of native beech wood with the anisotropic scattering of cellulose microfibrils at detector distances of 1 m (left) and 8 m (right) are shown below.

Figure 14. The measured water content after the pretreatment and the calculated, total water content. In comparison to the line of unity, the data shows good correlation demonstrating that water content was not changed during pretreatment.
